# Patients with mild traumatic brain injury and acute neck pain at the emergency department are a distinct category within the mTBI spectrum: a prospective multicentre cohort study

**DOI:** 10.1186/s12883-020-01887-x

**Published:** 2020-08-26

**Authors:** Sophie M. Coffeng, Bram Jacobs, Myrthe E. de Koning, Gerard Hageman, Gerwin Roks, Joukje van der Naalt

**Affiliations:** 1 Department of Emergency Medicine, University of Groningen, University Medical Center Groningen, Groningen, The Netherlands; 2 Department of Neurology, University of Groningen, University Medical Center Groningen, Groningen, The Netherlands; 3 Department of Neurology, Hospital Medisch Spectrum Twente, Enschede, The Netherlands; 4 Department of Neurology, Elisabeth Tweesteden Hospital Tilburg, Tilburg, The Netherlands

**Keywords:** Neck pain, Mild traumatic brain injury, Outcome, Posttraumatic complaints

## Abstract

**Background:**

Acute neck pain (ANP) has recently been demonstrated to be a predictor of persistent posttraumatic complaints after mild traumatic brain injury (mTBI). The aim of this study was to determine specific characteristics of patients with ANP following mTBI, their posttraumatic complaints and relationship with functional outcome.

**Methods:**

Data from a prospective follow-up study of 922 mTBI patients admitted to the emergency department (ED) in three level-one trauma centres were analysed. Patients were divided into two groups: 156 ANP patients and 766 no acute neck pain (nANP) patients. Posttraumatic complaints were evaluated 2 weeks and 6 months post-injury using standardized questionnaires and functional outcome was evaluated at 6 months with the Glasgow Outcome Scale Extended (GOSE)**.**

**Results:**

ANP patients were more often female (*p* < 0.01), younger (38 vs. 47 years, *p* < 0.01) with more associated acute symptoms at the ED (*p* < 0.05) compared to nANP patients. More motor vehicle accidents (12% vs. 6%, *p* = 0.01) and less head wounds (58% vs. 73%, *p* < 0.01) in ANP patients indicated ‘high-energy low-impact’ trauma mechanisms. ANP patients showed more posttraumatic complaints 2 weeks and 6 months post-injury (*p* < 0.05) and more often incomplete recovery (GOSE < 8) was present after 6 months (56% vs. 40%, *p* = 0.01).

**Conclusions:**

MTBI patients with acute neck pain at the ED constitute a distinct group within the mTBI spectrum with specific injury and demographic characteristics. Early identification of this at risk group already at the ED might allow specific and timely treatment to avoid development of incomplete recovery.

## Background

Traumatic brain injury (TBI) is one of the most common injuries seen at the emergency department (ED) worldwide with mild traumatic brain injury (mTBI) occurring in the majority of patients (80–90%) [1]. Posttraumatic complaints after mTBI including headache, dizziness, fatigue, irritability and concentration problems are a significant health problem. In earlier studies, various factors have been examined for the prediction of posttraumatic complaints. Injury characteristics and severity indices of mTBI were found not to be related to outcome whereas psychological factors such as pre-injury mental health status, emotional distress and maladaptive coping, were shown to have an association with the development of posttraumatic complaints and outcome [2–7].

Posttraumatic complaints after mTBI have originally been defined as a direct result of brain injury. However, in a recent prospective multicentre follow-up study, neck pain immediately after injury was found to be a novel predictive factor for incomplete recovery after mTBI [8]. This is congruent with the recent emerged view that concomitant injury to the cervical spine is contributing to on-going symptoms after sustaining a mTBI [9–11]. Additionally, neck pain is a common posttraumatic complaint after mTBI and in a follow-up study of mTBI after motor vehicle accidents neck pain was reported by 50% of patients 6 weeks after injury, even more frequently than complaints of headache [12]. The trauma mechanism of mTBI is frequently an acceleration-deceleration trauma and/or significant blunt impact to the head. For anatomical reasons it is plausible that there is concomitant cervical spine involvement with mTBI, resulting in complaints of neck pain [11]. Until now mTBI and cervical spine injury (without radiological and neurological signs) have been regarded as separate entities. However, posttraumatic complaints after mTBI and whiplash associated disorder after a flexion-extension injury of the cervical spine have corresponding features suggesting at least some interplay between head and spine injury [11–18].

Posttraumatic complaints after mTBI have a great impact on resumption of daily activities and quality of life of patients. Six months after injury 20–30% of the patients with mTBI have not resumed work and pre-injury activities [19, 20]. As a consequence the societal costs related to mTBI are substantial [21]. As mTBI is such a common injury, many neurologists will see patients with posttraumatic complaints in their work at the outpatient clinic. It is therefore important to find risk factors to identify patients at risk for posttraumatic complaints and incomplete recovery early after injury to provide adequate care and therapy. Since the first assessment is done at the ED, it is important to delineate mTBI with and without concomitant cervical spine injury at an early phase given the predictive role of acute neck pain for the development of posttraumatic complaints.

The goal of our study therefore is to elucidate the characteristics and course of acute neck pain accompanying mTBI and its relation to persistent posttraumatic complaints. In addition, we aimed to determine whether specific demographic, patient and trauma characteristics could be identified within the ANP patient category at the ED to formulate specific directions for clinical practice.

## Methods

### Aims of the study

The primary aim of the study was to describe the characteristics and course of acute neck pain accompanying mTBI and the relation to persistent posttraumatic complaints and functional outcome. In addition, the secondary aim was to determine if specific demographic and trauma characteristics could be identified directly at the ED or in the subacute phase 2 weeks post-injury in acute neck pain patients with incomplete recovery.

### Study design and setting

This study is part of a large prospective follow-up study (UPFRONT-study) conducted in three level-one trauma centres in The Netherlands [8]. The UPFRONT-study is registered in the University Medical Center Groningen research registry at the 6th of October, 2014 (research number 20140210).

All included patients received questionnaires at the ED, 2 weeks and 6 months post-injury. MTBI was defined as a head trauma with a Glasgow Coma Scale (GCS) score of 13–15 at the ED, posttraumatic amnesia of < 24 h and/or loss of consciousness of < 30 min [22]. Acute neck pain (ANP) after injury was defined as the presence of neck pain directly after the mTBI. This information was collected at the ED.

### Selection of participants

Patients with a mTBI and aged > 15 years were included at the ED from January 2013 to January 2015. Exclusion criteria were: time since trauma > 24 h, addiction to drugs or alcohol, severe co-morbidity, previous psychiatric disease warranting admission to a psychiatric department and the inability to follow-up (like language barrier or no permanent home address). Patients with a radiologic confirmed diagnosis of traumatic cervical spine injury were excluded, because these patients presumably have a different aetiology of neck pain. Additionally, patients who were not able to indicate the presence of acute neck pain (intubated at the ED or being too confused) and when neck pain was not documented in the hospital charts were excluded.

### Methods of measurement

Demographic data, injury related characteristics and information about hospital admission and discharge were collected from medical files. Pre-injury mental health problems were defined as psychiatric or psychological symptoms necessitating treatment by psychiatrist or psychologist or using psychotropic medication. A 7-point scale was used to define Dutch education level and years of education (YoE) (1 = primary school (< 6 YoE), 2 = finished primary school (6 YoE), 3 = did not finish secondary school (7–8 YoE), 4 = finished secondary school (9 YoE), 5 = finished secondary school (10–11 YoE), 6 = finished secondary school (12–16 YoE) and 7 = university degree (> 16 YoE)) [8]. For analysis we dichotomised education in low (score < 6) and high (score 6 and 7) educational level.

At the ED, a full neurological examination was performed by the resident neurology or emergency physician including the Glasgow Coma Scale (GCS). The Injury Severity Score (ISS) was based on data from hospital records. A head CT scan was performed during ED admission according to the local version of the CHIP-prediction rule [23]. Imaging of the cervical spine was done following the NEXUS criteria [24]. The imaging of the head and cervical spine was classified as normal or abnormal for this study.

### Questionnaires

Follow-up was done by questionnaires (by mail or web-based) sent to all UPFRONT participants at 2 weeks and 6 months after injury.

*Head Injury Symptom Checklist (HISC):* [25] The HISC contains 21 of the most common post-concussion symptoms. Pre-injury complaints and their severity levels 2 weeks and 6 months post-injury were evaluated as a score from 0 to 2 (0 = never, 1 = sometimes, 2 = often). Each separate posttraumatic symptom was corrected for the presence of pre-injury symptom level by subtracting the pre-injury score from the score 2 weeks or 6 months post-injury. The total amount of complaints (sum score 0–21) and severity (score 0–42) were calculated at each evaluation moment.

*Hospital Anxiety and Depression Scale (HADS):* [26] The HADS is a 14-item questionnaire to assess depression or anxiety complaints with two subscales for depression (HADS-D) and anxiety (HADS-A) of seven items each. Each item is rated on a scale of 0–3. A cut-off score of > 7 for each subscale is regarded to establish the presence of clinical depression or anxiety.

*Impact of Event Scale (IES):* [27] The IES is a self-report measure for assessment of posttraumatic stress and comprises 15 statements with scores ranging from 0 to 5. A cut-off score of 19 defines a posttraumatic stress disorder.

*Glasgow Outcome Scale – Extended (GOSE):* [28] Functional outcome at 6 months post-injury was determined by the GOSE. This structured questionnaire has an eight-point scale (1 = death, 2 = vegetative state, 3 = lower severe disability, 4 = upper severe disability, 5 = lower moderate disability, 6 = upper moderate disability, 7 = suboptimal recovery, 8 = complete recovery). Outcome was dichotomised as complete (GOSE = 8) or incomplete recovery (GOSE < 8).

### Primary data analysis

SPSS Data Editor 23.0 (IBM SPSS Statistics, SPSS inc., Chicago, IL) was used for statistical analysis. Differences in demographic and trauma characteristics between the ANP and nANP group were tested using two sample Student *t-*test for ISS score and nonparametric tests (Mann–Whitney U-test) for the rest of the characteristics as they were not normally distributed. The ISS score was presented as mean ± standard deviation, not normally distributed data as median [25th -75th percentile] and categorical variables as frequencies and percentages. Trauma mechanism (traffic accidents, falls and others) was used as one variable for univariate and multivariable analysis. Nominal statistics were performed with the Pearson *X*^2^ test. A two-tailed probability < 0.05 was considered to be significant. Binary logistic regression analysis was used to perform univariate and multivariable correlations to assess the risk factors of incomplete recovery in the ANP group. A 95% confidence interval (CI) was used for univariate and multivariable analysis. If variables were significant (*p* < 0.05) in univariate logistic regression analysis they were included in the multivariate binary logistic regression analysis. No other forms of variable selection were used for multivariable analysis.

## Results

### Characteristics of subjects

**Fig. 1:**
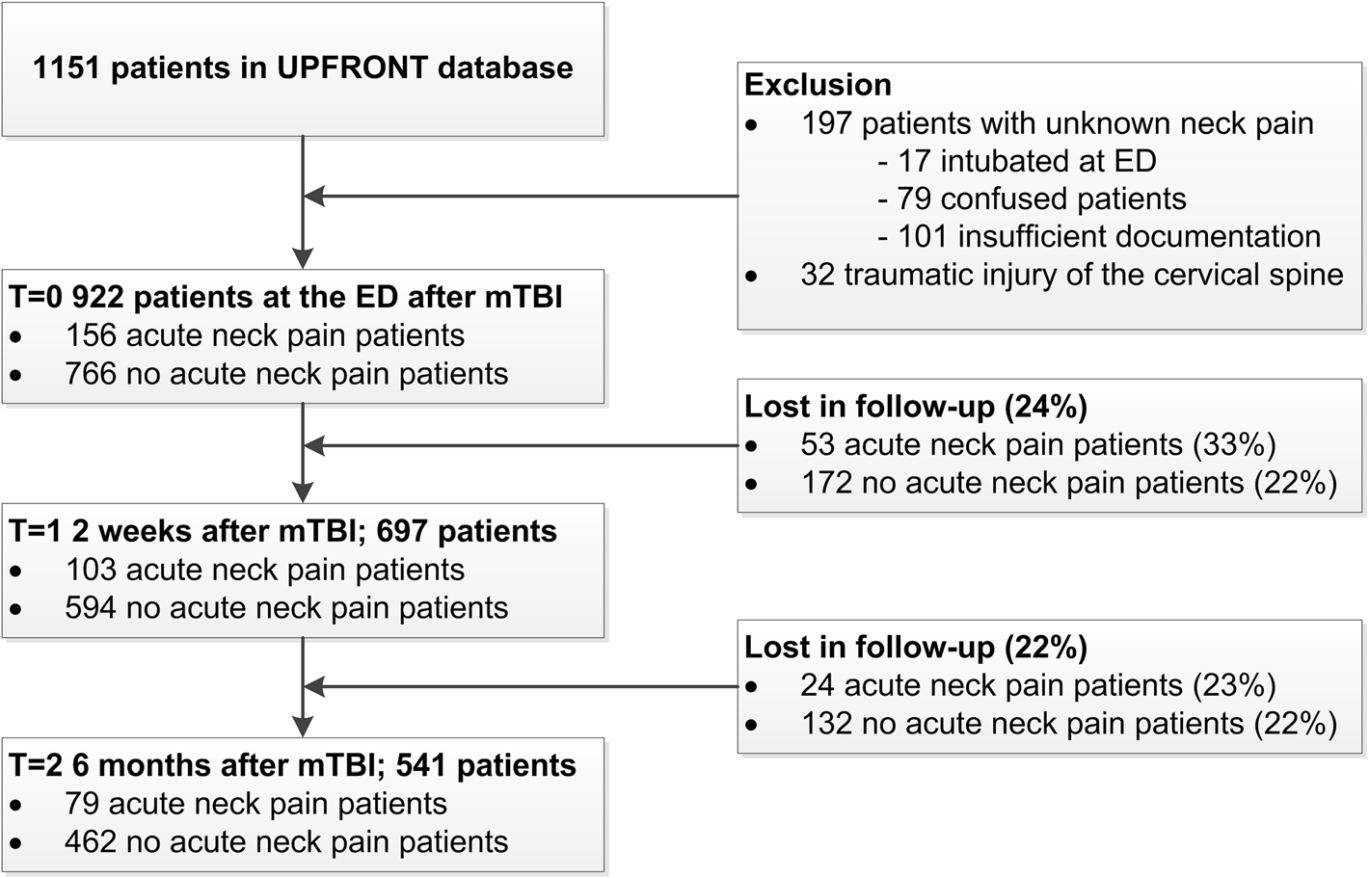
Flowchart Legend: ED: Emergency Department; mTBI: mild Traumatic Brain Injury

A total of 1151 patients was included in the UPFRONT-study. We excluded 32 patients with radiological cervical spine injury on CT and 197 patients with insufficient documentation of acute neck pain. Accordingly, data of 156 patients with acute neck pain (ANP) and 766 patients with no acute neck pain (nANP) accompanying mTBI were analysed. Of this group 12 ANP patients and 292 nANP patients did not undergo imaging of the cervical spine because they did not met the NEXUS criteria. In 19 patients data of cervical spine imaging were missing. After 2 weeks 24% and after 6 months 42% of the included patients was lost to follow-up (Fig. 1).

Table 1 shows the demographic characteristics for ANP patients versus nANP patients. ANP patients were significantly younger (38 [22–52] vs. 47 [25–62] (median ± [25th–75th percentiles]), *p* < 0.01) and more often female (51.9% vs. 35.4%, *p* < 0.01) compared to nANP patients. Pre-injury complaints, like headache (40.8% vs. 32.2%, *p* = 0.06), concentration problems (40.8% vs 36.0%, *p* = 0.63) and neck pain (28.7% vs. 21.3%, *p* = 0.06), were not significant different between ANP and nANP patients. Injury severity related parameters (GCS score, loss of consciousness and posttraumatic amnesia) were also comparable in both groups, except for the ISS score which was slightly lower in the ANP group (5.7 ± 3.1 vs. 7.0 ± 4.5 (mean ± standard deviation), *p* < 0.01) (Table 1). ISS scores of 92.3% of the ANP patients and 84.5% of the nANP patients were available. ANP patients had more acute complaints at the ED (Table 1). Patients with ANP showed fewer external head wounds (58.1% vs. 72.6%, *p* < 0.01) and the impact of the injury on the head was less frequently located on the frontal site of the head (34.6% versus 46.7%; *p* < 0.01). ANP patients were more often involved a motor vehicle accident compared to nANP patients (12.1% vs. 5.6%, *p* = 0.01) (Table 1).

**Table 1:** Demographic and injury characteristics of patients at the ED

	ANP patients *N* = 156	nANP patients *N* = 766	*P*-value
Gender (Female)	81 (51.9)	271 (35.4)	< 0.001
Age	38 (22–52)	47 (25–62)	< 0.001
Education level (low)^a^	49 (47.6)	314 (53.2)	0.290
Pre-injury mental health problems^a^	24 (21.8)	69 (11.6)	0.004
ISS^a^	6 (3.1)	7 (4.5)	0.001
GCS score ED			
*- 15*	110 (70.5)	514 (67.1)	0.406
*- 14*	37 (23.7)	203 (26.5)	0.470
*- 13*	9 (6.0)	46 (5.8)	0.910
Loss of consciousness	133 (85.1)	662 (86.4)	0.700
PTA > 1 h^b^	39 (26.0)	200 (27.1)	0.775
External head wounds^b^	86 (58.1)	547 (72.6)	< 0.001
Fractures of extremities	11 (7.3)	67 (8.9)	0.522
Head CT abnormalities^b^	14 (9.1)	92 (12.3)	0.265
Alcohol intoxication	46 (29.5)	285 (37.3)	0.064
Hospital admission (yes)	70 (44.9)	458 (59.8)	0.001
Symptoms at the ED			
*- Vomiting*^b^	20 (12.8)	93 (12.1)	0.814
*- Dizziness*^a^	30 (19.2)	81 (10.6)	0.002
*- Nausea*^b^	65 (41.7)	207 (27.0)	< 0.001
Trauma mechanism			
*- Traffic accidents*	36 (23.1)	154 (20.1)	0.403
*- motor vehicle accidents*	*19 (12.2)*	*43 (5.6)*	*0.004*
*- motorcycle*	*4 (2.6)*	*34 (4.4)*	*0.139*
*- bicycle*	*11 (7.1)*	*63 (8.2)*	*0.066*
*- pedestrian*	*1 (0.6)*	*7 (0.9)*	*0.203*
*- Falls*	97 (62.2)	509 (66.4)	0.306
*- Other*	23 (14.7)	103 (13.4)	0.667
*- violence*	*11 (7.1)*	*64 (8.4)*	*0.587*
*- sports*	*4 (2.6)*	*9 (1.2)*	*0.252*
*- not specified*	*8 (5.1)*	*30 (3.9)*	*0.488*

Data are n (%), mean (SD) or median (25th – 75th percentile). *ANP* acute neck pain post-injury, *nANP* No acute neck pain post-injury. Education level low = < 12 years of education, *ISS* Injury Severity Score, *GCS* Glasgow Coma Scale, *ED* Emergency Department, *PTA* Posttraumatic amnesia, Traffic accidents = all collisions by bicycle, motorcycle and car. ^a^ missing data 10–25%. ^b^ missing data < 10%, Two-sided *p* < 0.05 as criterion for significance was used

### Non-responders and excluded patients

49.3% Of the ANP group and 39.7% of the nANP group was lost to follow-up at 6 months post-injury. The non-responders were younger (30 [21–50] vs. 52 [34–64], *p* < 0.01) and had less frequent a maximal GCS score at the ED (29.7% vs. 36.0%, *p* = 0.04). Alcohol intoxication (42.1% vs 31.6%, *p* < 0.01) and violence (16.2% vs 2.4%, *p* < 0.01) were more frequent in the total non-responder group. ANP non-responders were younger compared to nANP non-responders (25 [19–40] vs. 32 [22–52], *p* = 0.001). For alcohol intoxication (*p* = 0.159), GCS scores (*p* = 0.466) and violence as trauma mechanism (*p* = 0.230) there were no differences between de ANP non-responders and nANP non-responders.

Patients that were excluded because of missing information on acute neck pain (*n* = 197) were relatively more severely injured: they had higher mean ISS (8.2 ± 5.6 vs. 6.8 ± 4.3, *p* < 0.01), less frequent a maximal GCS score at the ED (51.5% vs. 67.7% *p* < 0.01), more frequently PTA more than 1 h (43.3% vs. 26.9% *p* < 0.01) and more head CT scan abnormalities (22.6% vs. 11.7% *p* < 0.01).

### Posttraumatic complaints

**Fig. 2:**
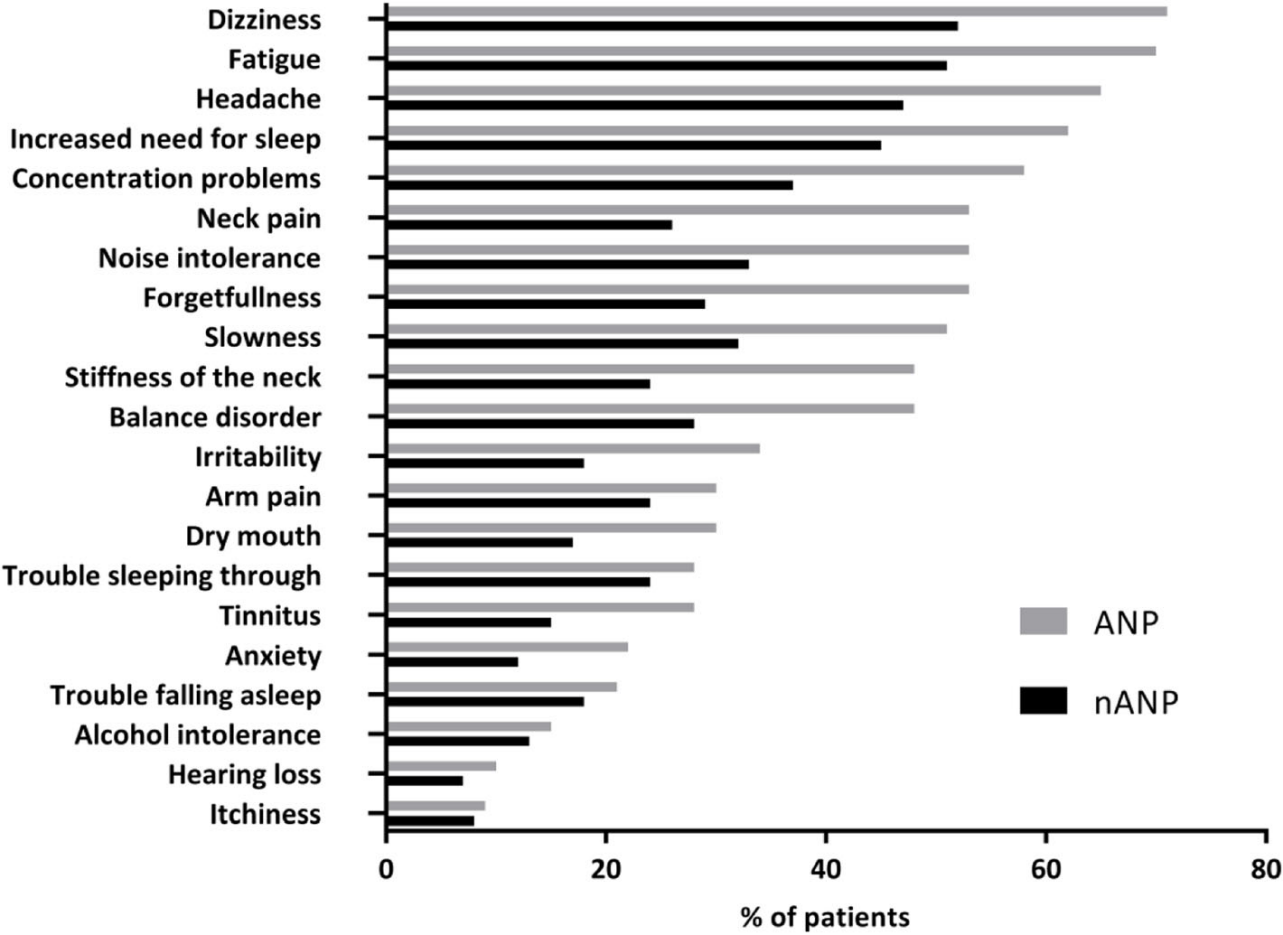
Posttraumatic complaints 2 weeks after mTBI. Legend: mTBI: mild Traumatic Brain Injury; ANP: Acute Neck Pain; nANP: no Acute Neck Pain

**Fig. 3:**
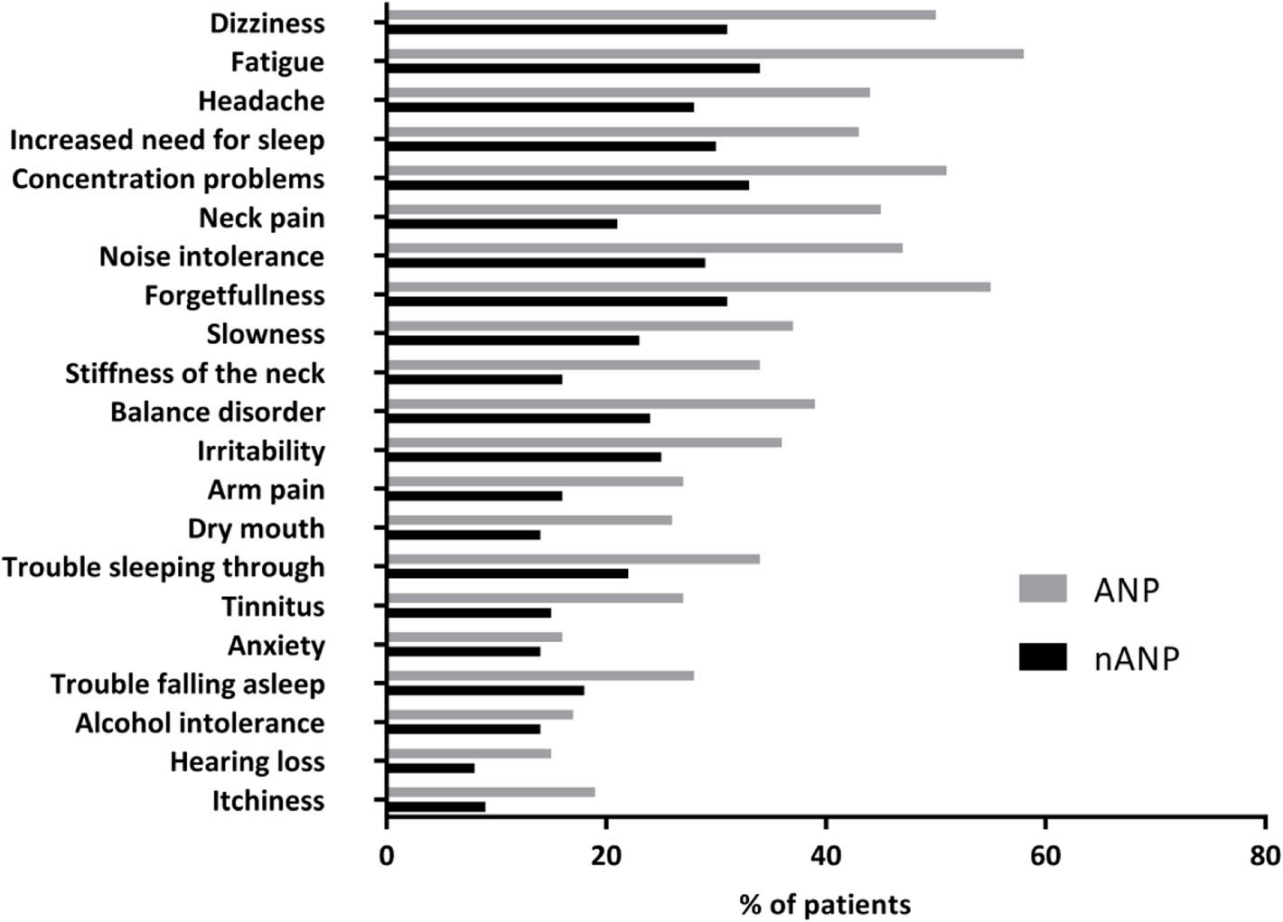
Posttraumatic complaints 6 months after mTBI. Legend: mTBI: mild Traumatic Brain Injury; ANP: Acute Neck Pain; nANP: no Acute Neck Pain

ANP patients had significantly more posttraumatic complaints compared to nANP patients with a sum score of 8 [5–11] vs. 4 [1–8] (*p* < 0.01) and a higher severity score of 10 [5–14] vs. 5 [1–9] (*p* < 0.01). Each specific complaint was also more frequent in the ANP group after 2 weeks (Fig. 2). Six months after injury ANP patients still had more posttraumatic complaints compared to nANP patients regarding the sum score (6 [0–11] vs. 2 [0–7], *p* < 0.01) and the severity score (7 [0–14] vs. 2 [0–8], *p* < 0.01). Also, each specific posttraumatic complaint was more frequent in the ANP group (Fig. 3).

### Emotional distress

Two weeks post-injury the percentage of patients with a HADS–A score indicative for anxiety (> 7) was significantly different between both groups (ANP 27.4% vs. nANP 16.3%, *p* = 0.01). There was no difference in frequency of depressive complaints between both groups on the HADS-D (> 7) (ANP 16.8% vs. nANP 15.3%, *p* = 0.70). The IES score indicative of emotional distress was significantly higher in the ANP group 2 weeks after mTBI (45.7% vs. 34.3%, *p* = 0.03).

### Functional outcome

Six months post-injury ANP patients had a poorer functional outcome compared to nANP patients: 55.7% vs. 40.0% showed an incomplete recovery (GOSE < 8) (*p* = 0.01). Table 2 shows the results of the univariate and multivariable analyses for incomplete recovery in the ANP group. Multivariable analysis showed that female gender (OR 2.94, 95% CI 1.13 to 7.64) and the absence of alcohol intoxication (OR 0.30, 95% CI 0.10 to 0.93) were correlated significantly with incomplete recovery in the ANP group.

**Table 2:** Univariate and multivariable analysis for GOSE < 8 in ANP patients

		ANP patients (***n*** = 79)					
		Univariate			Multivariable		
	Coding	OR	95% CI	***p***-value	OR	95% CI	***p***-value
Age	16–82	1.01	0.98–1.04	0.498			
Gender	Male (0)-Female (1)	3.21	1.27–8.12	0.014	2.94	1.13–7.64	0.027
Alcohol	No (0) – Yes (1)	0.27	0.09–0.80	0.019	0.30	0.10–0.93	0.037
Education level	Low (0) – High (1)	0.84	0.33–2.14	0.710			
GCS score ED	13–15	0.59	0.25–1.38	0.224			
ISS	2–25	1.19	0.97–1.45	0.090			
LOC	No (0) – Yes (1)	0.93	0.29–2.99	0.931			
PTA	< 1 h (0) - > 1 h (1)	0.82	0.29–2.31	0.703			
External head wounds	No (0) – Yes (1)	0.45	0.16–1.28	0.132			
Head CT abnormalities	No (0) – Yes (1)	1.22	0.31–4.71	0.777			
Hospital admission	No (0) – Yes (1)	1.01	0.41–2.48	0.977			
Acute complaints	No (0) – Yes (1)	1.33	0.44–4.00	0.608			
Trauma mechanism							
*- Traffic accidents*		4.00	1.18–13.6	0.033	NS	NS	NS
*- Falls*		0.25	0.07–0.85	0.026	NS	NS	NS
*- Other*		0.10	0.01–0.72	0.022	NS	NS	NS
Two weeks post-injury	No (0) – Yes (1)						
*- Anxiety*^*a*^		1.55	0.46–5.24	0.439			
*- Depression*^*a*^		3.73	0.73–19.1	0.114			
*- Posttraumatic stress*^*b*^		2.10	0.77–5.75	0.149			

Education level low = < 12 years of education, *GCS* Glasgow Coma Scale, *ED* Emergency Department, *ISS* Injury Severity Score, *LOC* Loss of consciousness, *NS* Not significant in multivariable analysis, *PTA* Posttraumatic amnesia, Acute complaints = headache, nausea, vomiting or dizziness at the ED, Traffic accidents = all collisions by bicycle, motorcycle and car. ^a^cut-off score of > 7 for anxiety or depression on the Hospital Anxiety and Depression Scale. ^b^A cut-off score of 19 on the Impact of Events Scale

## Discussion

This study shows that patients with acute neck pain accompanying mTBI constitute a distinct group with different demographic characteristics (female, younger age), with more acute complaints at the ED (nausea, headache, dizziness) and more frequently motor vehicle accidents as trauma mechanism, compared to those mTBI patients without acute neck pain. As acute neck pain is a risk factor for incomplete recovery we believe it is important to identify these patients as soon as possible, preferably already at the ED. [8] This early identification might provide the opportunity to start customized therapy at an early stage and contribute to the prevention of long-term post-traumatic complaints and unfavourable outcome.

The most important question of the current study was whether the group with ANP at the ED constitutes a distinct group that can be delineated from the broadly defined category of mTBI. One out of five patients with mTBI complains of neck pain at the ED. One of the more distinctive features of this patient group was the mechanism of injury. Interestingly, patients with ANP had less frequently external head wounds compared to the nANP group and the impact of the injury was less frequently located at the frontal site of the head. This may suggest a ‘high-energy low-impact’ trauma as trauma mechanism with involvement of a flexion-extension trauma of the cervical spine, in contrast to the nANP group suffering from a more direct head impact. Besides, ANP is often accompanied by complaints of dizziness and nausea and these symptoms are also associated with a cervical flexion-extension trauma [13–15]. Another point of view might be that patients with ANP are not a distinct category within the mTBI spectrum, assuming that acute neck pain is interrelated to the other acute complaints present at the ED after mTBI. Although ANP patients do have more nausea and vomiting than nANP patients at the ED, an earlier study from our research group showed that there was no correlation found of these complaints with incomplete recovery [8]. It is therefore unlikely that ANP is an indirect marker of other acute posttraumatic complaints, and ANP has to be regarded as an independent predictor for incomplete recovery after mTBI. In addition to this, incomplete recovery in ANP patients was only associated with female gender and the absence of alcohol intoxication. This may suggest that neck pain in itself is one of the main reasons why ANP patients did not completely recover.

The finding that alcohol intoxicated patients had a better functional outcome after mTBI is in accordance with results reported in previous research [2, 29]. An explanation for better recovery after alcohol intoxication might be that the emotional impact of the trauma was less severe, because the intensity of the experience was dampened by alcohol [30]. Also, patients with an acute alcohol intoxication could be initially judged to have more severe brain injury than was actually the case, due to depressant effect of alcohol on the central nervous system resulting in more favourable outcome. In addition, intoxicated patients in the mTBI literature are more often male and the trauma mechanism was more likely due to violence or falls [29]. This does not seem to correspond with the overall group of our ANP patients, that included more women and traffic accidents. Furthermore, female gender was also associated with incomplete recovery in the ANP group, which is consistent with previous mTBI research [31, 32]. It has been suggested that compared to men, women have increased pain sensitivity and an increased risk for clinical pain and are also more likely to report pain [33]. This might be one of the reasons why females with neck pain directly after a mTBI have a higher risk of incomplete recovery.

All patients in our population were initially treated as mTBI patients, but in case of ANP patients with a presumed additional cervical flexion-extension trauma mechanism other treatment at the ED and during follow-up might be necessary compared to nANP patients. The current treatment for patients with posttraumatic complaints after mTBI is to a large extent based on psycho-education and the advice to increase daily activities when symptoms improve [1]. However, ANP patients might benefit more from pain management and physical exercises in line with recent findings in sports medicine, where the involvement of the cervical spine in mTBI is thought to be a contributing factor to on-going symptoms [9–11]. These studies suggested that patients with neck pain after mTBI will benefit from specific therapy for the cervical spine. Exercise plays an important role in the management of these patients with neck pain after sport related mTBI, comparable to the approach of patients with whiplash associated disorder as opposed to current mTBI treatment [9, 10, 34–36]. However, consensus about the best treatment for mTBI patients with cervical tenderness is lacking and all different kinds of (physical) therapy are advised. Most patients in these studies were treated with a combination of these therapies, for example manual therapy, stability therapy, active release therapy or spinal manipulative therapy, with varying effect [37].

In our study, we found that ANP patients show a different prevalence of several posttraumatic symptoms compared to nANP patients: mTBI patients with acute neck pain display more physical problems, stress and anxiety 2 weeks post-injury compared to mTBI patients without acute neck pain. As acute neck pain is an independent risk factor for incomplete recovery, which can already be identified at the ED, it provides the opportunity to start early and targeted therapy for this specific category of mTBI patients to avoid incomplete recovery.

Although this study identified specific characteristics for patients with acute neck pain accompanying mTBI, some limitations should be mentioned. Firstly, from a substantial number of patients no clear information about the presence of acute neck pain was noted in the medical charts. This could be partly explained because half of these patients were confused or were intubated at the ED limiting documentation. However, for the current study we were primarily interested in patients who could indicate their posttraumatic complaints at the ED to explore the characteristics of the ANP group [8]. Secondly, there was loss to follow-up after 2 weeks and 6 months after injury. This might have resulted in a bias towards worse outcome, as patients without posttraumatic complaints might be difficult to motivate for follow-up [38]. Nevertheless, the dropout in our study is comparable with other longitudinal mTBI follow-up studies facilitating comparison of results [2, 19]. Although the dropout in the nANP group was larger compared to the dropout in the ANP group, both dropout groups were comparable except for their age. Thirdly, some demographic and trauma characteristics were not complete for all patients because they were derived from medical reports, in particular the presence of pre-injury mental health problems and this aspect should thereby interpret with caution. Lastly, the GOSE is a scale to asses functional outcome in TBI. However, it does not distinguish changes in daily functioning of the patient due to the TBI or due to other injuries of the body [28]. Therefore, it may be possible that in some cases with multiple extracranial injuries the determined functional outcome may be due to other injuries, although the overall rate of extracranial injuries in the ANP and nANP group was low. Despite these aforementioned limitations, we think that this study comprises a substantial sample to emphasize our finding that ANP is a predictor for long-term incomplete recovery after mTBI and patients with ANP are a specific patient category within the mTBI spectrum that should be recognized at the ED in order to provide early and targeted therapy.

## Conclusions

In summary, patients with acute neck pain accompanying mTBI have a higher risk for incomplete recovery with more posttraumatic complaints. Involvement of flexion-extension trauma of cervical spine is found to be a contributing factor to on-going symptoms which suggests more high-energy low-impact traumas in this patient group. As mTBI with posttraumatic complaints are commonly encountered in daily practice, it is important to make neurologists and ED physicians more aware of the clinical spectrum of mTBI with distinct patient groups, in particular patients with accompanying acute neck pain. Those patients can already be identified at the ED enabling to start first treatment already at the ED to avoid the development of persistent complaints and incomplete recovery. Further research is necessary in this specific category of patients to investigate what kind of treatment is most suitable to improve posttraumatic complaints and the functional outcome.

## Data Availability

The datasets generated and/or analysed during the current study are not publicly available due to no specific consent from subjects, but are available from the corresponding author on reasonable request.

## Abbreviations

ANP: Acute Neck Pain
ED: Emergency Department
GCS: Glasgow Coma Scale
GOSE: Glasgow Outcome Scale Extended
HISC: Head Injury Symptom Checklist
HADS: Hospital Anxiety and Depression Scale
IES: Impact of Event Scale
ISS: Injury Severity Score
mTBI: Mild Traumatic Brain Injury
nANP: No Acute Neck Pain
TBI: Traumatic Brain Injury
YoE: Years of Education
